# Anatomic medial patellofemoral ligament (MPFL) reconstruction with and without tibial tuberosity osteotomy for objective patellar instability

**DOI:** 10.1007/s12306-021-00721-y

**Published:** 2021-07-10

**Authors:** A. Pautasso, L. Sabatini, M. Capella, F. Saccia, L. Rissolio, G. Boasso, F. Atzori, A. Massè

**Affiliations:** 1 University of Turin, Via Gianfranco Zuretti 29, 10126 Turin, Italy; 2 Orthopaedic and Traumatology Department, Orthopaedic and Trauma Center, University of Turin, Via Gianfranco Zuretti 29, 10126 Turin, Italy; 3 Orthopaedic and Traumatology Department, Koelliker Hospital, Corso Galileo Ferraris 247, 10134 Turin, Italy; 4 Orthopaedic Department, Piccola casa della Divina Provvidenza, Via San Giuseppe Benedetto Cottolengo, 10152 Turin, Italy

**Keywords:** MPFL reconstruction, Schӧttle technique, Objective patellar instability, Tegner Activity Scale

## Abstract

**Purpose:**

The aim of this study was to evaluate the clinical outcomes of patients treated with anatomic medial patellofemoral ligament (MPFL) reconstruction with and without tibial tuberosity osteotomy (TTO). Correlations between patient's age, gender, pre-injury physical activity and the achieved results were investigated as secondary endpoints.

**Methods:**

An observational retrospective study with prospective collected data was performed. Inclusion criteria were: treatment with anatomic MPFL reconstruction with gracilis tendon according to Schӧttle’s technique performed between 2011 and 2017; associated TTO as unique accessory procedure; skeletal joint maturity; a minimum follow-up of 12 months after surgery. Clinical outcomes were assessed with the Kujala, Lysholm and Tegner scores.

**Results:**

Forty patients (42 knees) were included, 64% of them underwent TTO. The Kujala score significantly improved from 47.4 ± 17.6 preoperatively to 89.4 ± 13.6 postoperatively (*p* < 0.01). The average Lysholm score was 45.6 ± 20.5 preoperatively: it showed a significant increase to 89.8 ± 12.8 postoperatively (*p* < 0.01). Pre-injury mean Tegner was 5.9 ± 1.8, while it dropped to 3.0 ± 1.6 after injury. After surgery, Tegner resulted 4.9 ± 1.6. Forty-three percent of patients regained the pre-injury sport activity level. Redislocation rate was 2.4%.

**Conclusion:**

Anatomic MPFL reconstruction allows excellent patellar stability recovery, knee functionality improvement, return to Activities of Daily Living and a low redislocation rate. Better results were achieved in younger (under 30 years old) and higher sports activity-level subjects. The TTO association provided clinical results comparable to isolated MPFL reconstructions, suggesting that the two procedures can be safely accomplished together without affecting the positive outcomes.

**Level of evidence:**

Level IV.

## Introduction

Acute patellar dislocations represent the 2–3% of all knee injuries [1], with an incidence of 6/100.000 person-year in the overall population [2]. It affects mostly young and active people with peak incidence among females between 10 and 17 years old. Recurrence rates of 17–49% were reported after the first episode of dislocation, with higher values among patients under 20 years old [3]. Recurrence rates increase up to 44–71% after the second episode of dislocation. Patellar dislocation usually occurs laterally, and an associated osteochondral lesion is present in 49% of patients; medial patellofemoral ligament (MPFL) disruption occurs in 90–100% of patellar dislocations [4].

Patients who have experienced at least one episode of patellar dislocation in their life present one of the main factors of instability: (1) trochlear dysplasia [5]; (2) patella alta [6]; (3) pathologic tibial tuberosity–trochlear groove (TT-TG) distance [7, 8].

The correct treatment strategy for the first episode of lateral patellar dislocation is still controversial. Conservative treatment is still widely adopted, but it is burdened by high rates of dissatisfaction: about 58% of patients report relevant limitations in physical activities at 6 months after injury and 55% of these do not return to the same pre-injury activity level [9]. Smith et al. [10] showed a lower recurrence rate for surgical-treated patients, but a greater incidence of patellofemoral osteoarthritis. In their Cochrane Review [11], investigating the benefits of surgical treatment versus conservative approach in 339 patients with 2–7 years’ follow-up, the same authors concluded that the evidences were not enough to establish a significant supremacy of one treatment rather than the others for the first patellar dislocation. Recently, a systematic review by Erickson et al. [12] including four meta-analyses pointed out that the surgical treatment of acute patellar dislocations resulted in lower recurrence rates (24% vs 36% of conservative treatment) even if the functional scores did not improve.

Conservative therapy involves the use of a brace or taping techniques in addition to the gradual recovery of full mobility and muscles strength before returning to sports [13, 14].

Surgical treatment for patellar dislocation is usually strongly suggested in case of symptomatic bone fragments or osteochondral lesions, failure of conservative therapy, high risk of recurrence and recurrent patellar dislocations with low quality of life [15–18]. Many surgical procedures are suggested in the literature: acute MPFL repair; anatomic MPFL reconstruction; tibial tuberosity osteotomy (TTO); trochleoplasty; femoral derotation osteotomy; lateral release; VMO plasty.

The aim of this retrospective study was to evaluate the clinical outcomes of patients treated with anatomic MPFL reconstruction according to Schӧttle’s surgical technique with and without TT osteotomy. Functional scores, redislocation rate and return to Activities of Daily Living (ADL) and sport activities have been assessed. Correlations between the patient’s age, gender, pre-injury physical activity and the achieved results were investigated as secondary endpoints. Finally, the outcomes of the patients undergoing the isolated MPFL reconstruction have been compared with the ones of patients undergoing the same procedure in addition to TTO.

## Material and methods

An observational retrospective study with prospective collected data was conducted.

Inclusion criteria were: chronic objective patellofemoral instability treated with anatomic MPFL reconstruction with autologous gracilis tendon according to Schӧttle’s technique between 2011 and 2017 at CTO Hospital—Città della Salute e della Scienza (Turin); first (with an important apprehension sign) or recurrent episodes of patellar dislocation; failed conservative treatment; associated TTO as unique accessory procedure; absence of secondary further lesions inside and around the knee (e.g., osteochondral lesions, meniscal ruptures, etc.); skeletal joint maturity; a minimum follow-up of 12 months after surgery.

Exclusion criteria were: MPFL reconstruction not performed according to Schӧttle’s technique; any associated procedure other than TTO like trochleoplasty, ACL reconstruction, meniscectomy or meniscal suture; revision procedures for patients already surgically treated in the past for patellar instability; lack of complete clinical and radiological records.

All patients were mandatory studied with preoperative radiological analysis: X-ray, CT scan and MRI. Caton—Deschamps Index (CDI), patellar tilt and TT-TG were evaluated as surgical planning. In case of CDI > 1.2 and/or TT-TG > 20 mm, a tibial tuberosity transposition procedure was performed in addition to MPFL reconstruction.

The anatomic MPFL reconstruction was performed with gracilis tendon autograft according to Schӧttle. All procedures started with routine arthroscopic evaluation followed by gracilis tendon harvesting. The graft loop was fixed to the medial edge of the patella with two anchors (BioComposite™ SwiveLock® 4.75 mm, Arthrex, Naples—Florida, USA) to create a double-bundle construct, while the two tails of the graft were secured in the femoral socket at the Schӧttle point [19] with an interference screw (Genesys™ Matryx®, ConMed Linvatec, Largo—Florida, USA) at 20 degrees of knee flexion.

After surgery, all patients wore a long-leg hinged knee brace with 0°–30° of knee flexion for two weeks and then 30° of knee flexion increase weekly. Weight bearing as tolerated with crutches was allowed to isolated MPFL reconstructions, while patients with associated TTO walked with crutches up to 40 days after surgery. Physiotherapy started from 4th week: isometric and isokinetic muscle reinforcement with closed-chain exercises was performed until 10th week; then, static proprioceptive and neuro-muscular control exercises were added, with transition to dynamic proprioceptive exercises within 20th week. Patients who underwent TTO were radiographically monitored until complete healing. Usually, X-rays controls (antero-posterior and lateral projections) were performed in the immediate postoperative time**,** 30 days, 3 months and 6 months after surgery.

Patellar dislocation rate, mechanism of injury and redislocation rate after surgery were recorded. Functional results were evaluated using the Kujala and Lysholm score before and after surgery. These scales are specific to evaluate femoral–patellar joint through different questions concerning the normal or pathologic gait, the use of crutches, the presence of swelling and/or pain during the day, the atrophy of the thigh, the knee flexion deficit and the feeling of patellar instability. A score from 0 to 100 is given on both questionnaires.

The sport activity levels were evaluated using the Tegner Activity Scale: this one assigns a score from 0 to 10 (0—disabled subject due to knee pathology; 10—subject who participates in national or international competitions of contact sports, e.g., football, rugby, etc.) based on daily or sporting activity that patient is able to perform, respectively, during the pre-injury, preoperative period and at the time of follow-up after surgery.

### Demographic data

Forty-five patients (47 knees) with objective patellar instability were treated. Five patients were excluded: in 2 cases an ACL reconstruction was associated with MPFL reconstruction, while in the other 3 cases the MPFL reconstruction was associated with a deepening trochleoplasty.

According to inclusion and exclusion criteria, 40 patients (42 knees, Table 1) were eligible for the study; 28 (70%) were females. The mean age of the patients at the time of surgery was 24 ± 9 (range, 14–46) years, and the average follow-up was 34 ± 22 (range, 12–85) months.

**Table 1:** Summary statistics of study population

Patients (*n*) Knees treated (*n*)	40 42
Female (*n*, %)	28 (70%)
Male (*n*, %)	12 (30%)
Mean age at surgery (years old)	24 ± 9 (14–46)
Mean age at follow-up (years old)	27 ± 9 (15–48)
Mean follow-up (months)	34 ± 22 (12–85)
Patients younger than 30 y.o. (*n*, %)	31 (77.5%)
Patients older than 29 y.o. (*n*, %)	9 (12.5%)
Right (*n*)	16
Left (*n*)	26
Associated TTO (*n*, %)	27 (64%)
Medialization according to Elmslie–Trillat (*n*)	10
Distalization (*n*)	8
Medialization plus distalization (*n*)	9
Isolated MPFL reconstruction (*n*, %)	15 (36%)
Pre-surgery dislocations rate	
1 episode (*n*, %)	3 (7%)
2 episodes (*n*, %)	13 (33%)
3 episodes (*n*, %)	5 (12%)
4 episodes or more (*n*, %)	19 (48%)
Dislocation mechanism	
During daily activities (*n*, %)	32 (80%)
During sport activities (*n*, %)	8 (20%)

Thirty-seven patients (93%) suffered multiple dislocations before surgery, while only three of them (7%) had only one dislocating event with important apprehension sign. In 32 (80%) patients, patellar dislocation usually occurred during daily activities, while in the others only during sport practice.

According to clinical and radiological findings, TTO was indicated in 27 knees (64%): an Elmslie–Trillat technique was performed in 10 cases, a distalization in 8 cases and a distalization plus medialization in 9 cases. For the remaining 15 cases (36%), isolated anatomic MPFL reconstruction was considered sufficient to provide proper patellar stabilization.

## Statistical methodology

The results achieved were statistically analyzed using MedCalc® software. A Kolmogorov–Smirnov test was used to preliminary assess the variable distribution in all data series; then, Student’s *t*-test was used to compared Kujala, Lysholm and Tegner results before and after surgery. Moreover, a multiple regression was performed in order to analyze the influence of Tegner pre-injury, age and gender on Tegner post-surgery. Student’s *t*-test and Mann–Whitney test were, respectively, used to compare outcomes between patients younger or older than 30 and between males and females. Finally, the outcomes of isolated MPFL reconstructions were compared with those associated with TTO through Student’s *t*-test.

## Results

### Outcomes

**Fig. 1:**
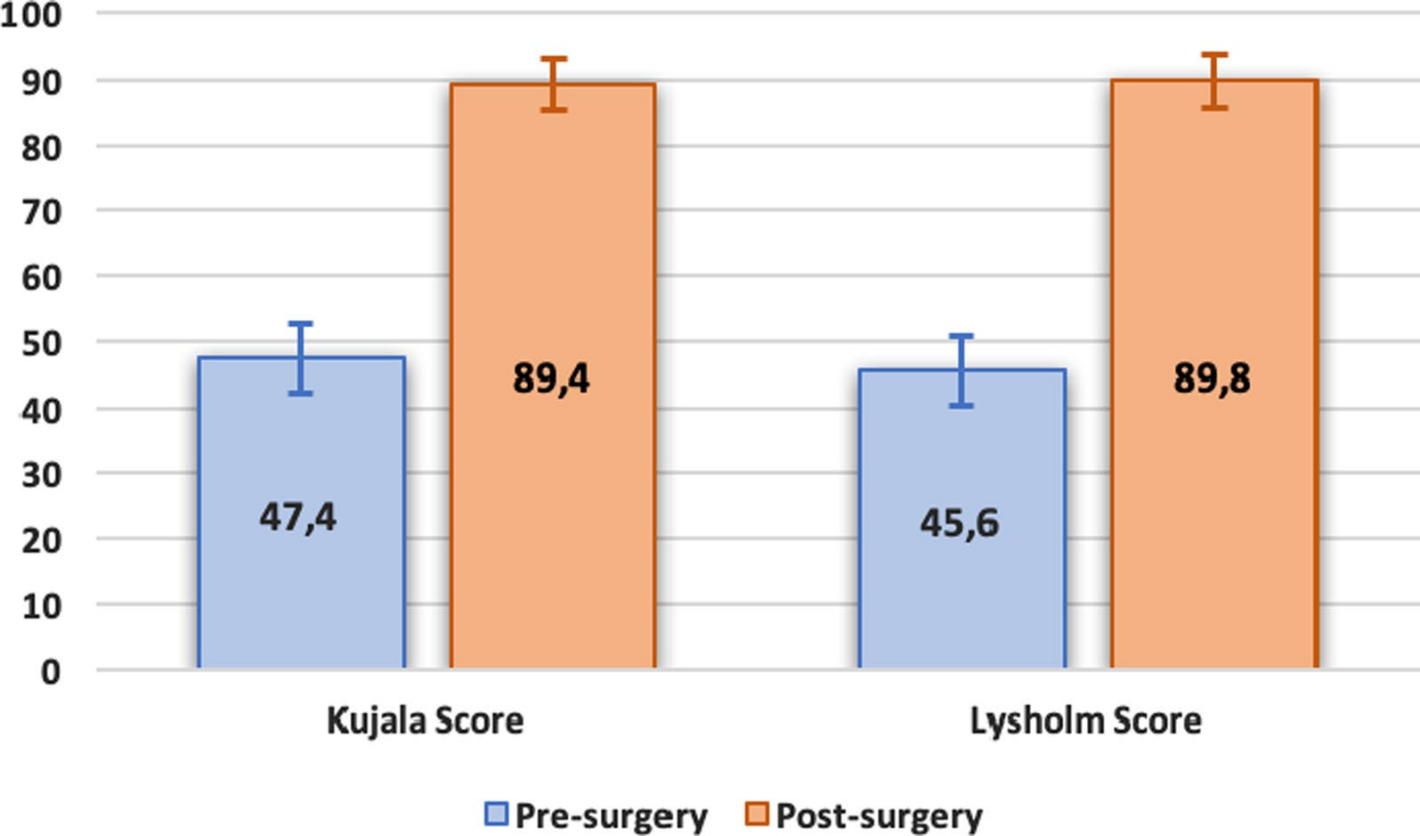
Kujala and Lysholm scores pre- and post-surgery (mean increase in Kujala score: 42.0, *p* < 0.01; mean increase in Lysholm score: 44.2, *p* < 0.01)

The Kujala score significantly increased (paired sample Student’s *t*-test *p* < 0.01) from an average of 47.4 ± 17.6 (range**,** 7.0–81.0) preoperatively to an average of 89.4 ± 13.6 (range**,** 42.0–100.0) postoperatively; in only two patients these results were below 60 points at the follow-up (Fig. 1, Table 2).

**Table 2:** Summary pre- and post- surgery scores results

	Pre-injury	Pre-surgery	Post-surgery
Kujala score	/	47.4 ± 17.6 (7.0–81.0)	89.4 ± 13.6 (42.0–100.0)
Lysholm score	/	45.6 ± 20.5 (4.0–86.0)	89.8 ± 12.8 (41.0–100.0)
Tegner score	5.9 ± 1.8 (2.0–9.0)	3.0 ± 1.6 (1.0–7.0)	4.9 ± 1.6 (2.0–7.0)

The average Lysholm score significantly increased (paired sample Student’s *t*-test; *p* < 0.01) from 45.6 ± 20.5 (range**,** 4.0–86.0) to 89.8 ± 12.8 (range, 41.0–100.0). Patients gained an average of 44.2 points.

**Fig. 2:**
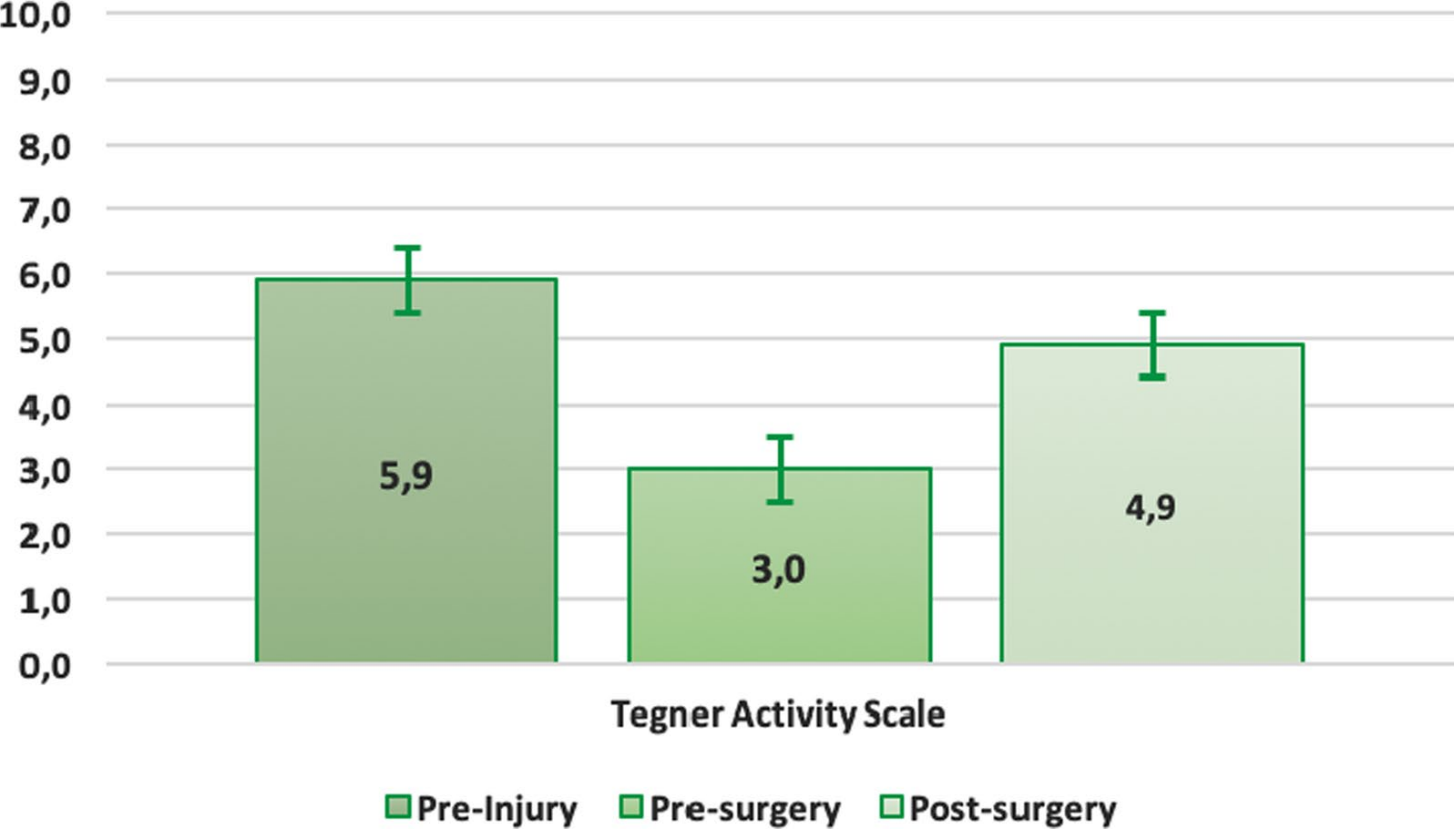
Tegner scores pre-injury, pre- and post- surgery (mean increase in Tegner pre- to post-surgery: 1.9, *p* < 0.01; mean difference between Tegner post-surgery and pre-injury: − 1.1, *p* < 0.01)

The Tegner score was recorded before the injury (mean value 5.9 ± 1.8; range, 2.0–9.0), before surgery (mean value 3.0 ± 1.6; range, 1.0–7.0) and after surgery (mean value 4.9 ± 1.6; range, 2.0–7.0). The mean postoperative activity-level score was significantly higher than the preoperative score (average gain of 1.9, paired sample Student’s *t*-test *p* < 0.01), but it was still significantly lower than pre-injury score (average loss of 1.1, paired sample Student’s *t*-test; *p* < 0.01) (Fig. 2, Table 2). Indeed, at the last follow-up only 17 patients (43%) returned to the same sport activity they used to practice before injury.

### Patient-related factors

In a multiple regression analysis, pre-injury activity level, age and gender were tested toward functional post-surgery outcomes; hence, using the linear regression models while excluding the parameters "age" and "gender" (*p* = 0.21 and *p* = 0.16, respectively), the resulted post-surgery functional scores' values were significantly impacted by the Tegner activity-level pre-injury scores (Kujala: *p* = 0.03; Lysholm: *p* = 0.04; Tegner: *p* < 0.01).

No significant differences were found between gender in post-surgery Kujala and Lysholm score**s** (independent sample Student’s *t*-test; *p* = 0.99 and *p* = 0.95, respectively); however, males reached a higher Tegner activity-level score after surgery (*p* = 0.03), as they started with higher values before injury (*p* = 0.03).

**Fig. 3:**
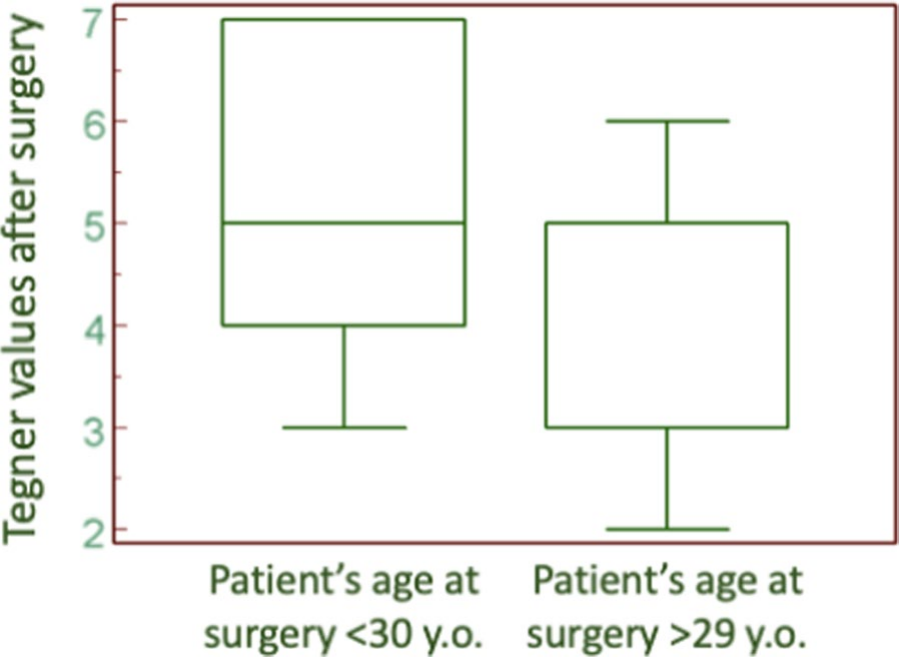
Tegner score post-surgery comparison between patients younger and older than 30 years old (Mann–Whitney’s test, *p* = 0.01)

In a linear regression model, increasing age was significantly related to a decline in functional scores (*p* = 0.01). Furthermore, evaluating patients under and above 30 years old, a difference in Tegner Scale was found both before and after surgery (Fig. 3): younger patients had a better return to sport and high demanding activities (Mann–Whitney’s test: *p* = 0.01). However, there was no statistically significant difference in Kujala and Lysholm scores between patients younger and older than 30 years old (Mann–Whitney’s test: *p* = 0.08 and *p* = 0.06, respectively).

### Associated Tibial Tuberosity Osteotomy (TTO)

Patients who underwent isolated MPFL reconstruction (15 knees—1st group) were compared to those who underwent associated TTO (27 knees—2° group). The mean age at the time of surgery was 27.6 ± 5.9 in the 1st group and 21.5 ± 2.9 in the 2nd group. The average Kujala, Lysholm and Tegner scores before surgery in the 1st group were: 47.8 ± 8.3; 48.3 ± 9.5 and 3.3 ± 0.8, respectively, while in the 2nd group they were: 47.1 ± 7.6, 44.1 ± 8.9 and 2.9 ± 0.7, respectively. No statistically significant difference was detected between the two groups in any score before surgery (Independent samples Student’s *t*-test, Kujala: *p* = 0.78, Lysholm: *p* = 0.16, Tegner: *p* = 0.10). The average Kujala, Lysholm and Tegner scores post-surgery were similar too, without significant statistical differences (independent sample Student’s *t*-test, results shown in Table 3).

**Table 3:** Descriptive statistics of the subgroups of patients who underwent isolated MPFL reconstruction or associated with TTO, with comparison between their outcome scores (NS: not significant)

	Isolated MPFL reconstruction	MPFL reconstruction with TTO	Δ Difference
Number of treated knees	15	27	
Mean age at surgery (years old)	27.6 ± 5.9 (14–45)	21.5 ± 2.9 (15–45)	
Mean age at follow-up (years old)	30.4 ± 6.0 (15–47)	24.5 ± 3.1 (16–48)	
Mean follow-up (months)	32.6 ± 11.7 (12–79)	35.4 ± 9.1 (12–85)	
Kujala post-surgery	91.1 ± 14.0 (44.0–100.0)	88.5 ± 13.5 (42.0–100.0)	2.6 (NS)
Lysholm post-surgery	90.1 ± 14.8 (41.0–100.0)	89.6 ± 11.9 (61.0–100.0)	0.5 (NS)
Tegner post-surgery	4.8 ± 1.4 (3.0–7.0)	4.9 ± 1.7 (2.0–7.0)	-0.1 (NS)

### Complications

There was only one case (2.4%) of patella redislocation after isolated MPFL reconstruction. Within the remaining 41 patients, 40 were satisfied after the surgical treatment performed while one patient complained about anterior knee pain and dissatisfaction. There were no cases of infections, wound breakdown, deep vein thrombosis or osteotomy healing concerns.

## Discussion

The purpose of this study was to evaluate the clinical outcomes of patients treated with anatomic MPFL reconstruction and their relations with patient’s age, gender and pre-injury activity level. Furthermore, the clinical and functional outcomes of patients undergoing the isolated anatomic MPFL reconstruction were compared with the ones of patients undergoing the same procedure in addition with TTO.

As highlighted in the previous literature, the results of this study support the key role of the MPFL reconstruction in patellar stabilization [20]: after surgery, a high rate of patient satisfaction and a significant increase in every functional score were obtained. In our series, Kujala score gained 42.0 points from a mean of 47.4 ± 17.6 to a mean of 89.4 ± 13.6 after surgery. Mackay et al. [21] in their systematic review analyzed different techniques for MPFL reconstruction, and they found an overall improvement in Kujala score of 36 points (from 51.6 [IC95%: 46.71–56.49] to 87.77 [IC95%: 85.15–90.39]). Mulliez et al. [22] evaluated 129 knees with MPFL reconstruction performed with Schӧttle’s technique (38 ligament reconstructions were associated with TTO procedures): they had an average increase in Kujala score of 21.2 points, from 53.5 ± 22.7 preoperative to 74.7 ± 20.5 postoperative.

About the sport**s** activity levels, our scores were comparable to the findings of Zhao et al. [23], who evaluated the return to sport in 45 patients after a follow-up of 60 months and reported an increase of 2.6 points in Tegner activity-level scale with a final value of 5.7 ± 1.7 after surgery. Although our patients had an increase from 3.0 ± 1.6 before surgery to 4.9 ± 1.6 after surgery, they did not reach the average pre-injury level of 5.9 ± 1.8; the patients with higher pre-injury Tegner scores reached better post-surgery results.

However, all patients except one were able to perform at least slight physical activities (Tegner Activity Scale 3): the same level of pre-injury sport activity was regained from 43% out of all the subjects at the last follow-up.

Similarly, Damasena et al. [24] and Mykashima et al. [25] in their papers reported that the return to sport at the same level is often an issue: they, respectively, found that only 10% and 54% of patients returned to play at the same level as before their injury.

About the correlation between gender and post-surgery outcomes, this was not statistically significant in terms of Kujala and Lysholm scores (*p* = 0.99 and *p* = 0.95, respectively). The only difference in favor of males regarded Tegner postoperative scores, biased by higher pre-injury activity levels in males than females (*p* = 0.03) (Table 3).

Stratifying by age, younger patients showed a better return to sport and higher outcome scores (Fig. 3). Enderlein et al. [26], in their prospective study on 240 MPFL reconstructions, underlined as an age above 30 years old could negatively affect the outcomes; this may be attributed to a greater dislocations rate, resulting in a more significant chondral damage. In our study, patients younger than 30 years similarly obtained slightly higher Kujala and Lysholm scores, while they reached a statistically significant advantage in terms of post-surgery Tegner scores.

At the end, we found no statistically significant difference in functional outcomes between isolated MPFL reconstructions and those associated with TTO. Watanabe et al. [27] reported a mean postoperative Lysholm score of 92.4 ± 7.6 among 29 isolated MPFL reconstructions and of 89.6 ± 11.1 among 13 MPFL reconstructions associated with TTO. Similar results emerged from our study with an average Lysholm score of 90.1 ± 14.8 and 89.6 ± 11.9, respectively, with no significant differences in terms of complication rates. This suggests that in patients with clinical and anatomic features (CDI > 1.2 and/or TT-TG > 20 mm), the two surgical procedures can be safely accomplished together during the same surgical intervention in order to restore patellar stability without the risk of functional impairment or increased complication rate.

In the current study, we had only one recurrence of dislocation (2.4%), in our first patient treated with Schöttle’s technique. This adverse event could be probably attributed to a technical mistake in the first part of our learning curve. However, the overall redislocation rate is the same observed by Mackay et al. [21] in their systematic review (2.4%, IC95%: 1.29–4.46).

### Limitations and strengths of the study

In conclusion, this study has several limitations. Firstly, an intrinsic limitation is due to the retrospective setting of the study; secondly, we reported a small sample, especially after stratification, and lastly, our follow-up is too short to consider further aspects and complications such as the degree of osteoarthritis eventually induced by surgical procedures.

However, the stringent inclusion criteria and the scrupulous methodology used to compare different groups of patients underwent to MPFL reconstruction gave to this work importance in terms of obtained results. This surgical technique provided interesting and comforting results in according with those present in the recent literature. Further studies are needed along this path.

## Conclusions

Anatomic MPFL reconstruction with gracilis tendon autograft according to Schöttle’s technique provides an excellent recovery of patellar stability with a low redislocation rate, a significant improvement in knee function and a predictable return to ADL in our series. Almost half of the patients regained the pre-injury sport activity level, consistently with the results reported in the literature. Higher activity levels were achieved by younger and fitter subjects, while patient’s gender did not affect the outcomes. MPFL reconstructions associated with TTO provided clinical results comparable to isolated MPFL reconstructions, suggesting that the two procedures can be safely accomplished together whenever needed without affecting the positive outcomes.
